# Ablation of Red Stable Transfected Claudin Expressing Canine Prostate Adenocarcinoma and Transitional Cell Carcinoma Cell Lines by C-CPE Gold-Nanoparticle-Mediated Laser Intervention

**DOI:** 10.3390/ijms222212289

**Published:** 2021-11-13

**Authors:** Suhayla Alnajjar, Ingo Nolte, Annegret Becker, Jan Torben Schille, Nares Trakooljul, Marcus Frank, Anaclet Ngezahayo, Hugo Murua Escobar

**Affiliations:** 1 Small Animal Clinic, University of Veterinary Medicine Hannover, 30559 Hannover, Germany; suhayla.alnajjar@tiho-hannover.de (S.A.); ingo.nolte@tiho-hannover.de (I.N.); jan.torben.schille@tiho-hannover.de (J.T.S.); 2 Division of Medicine, Haematology, Oncology and Palliative Medicine, University of Rostock, 18057 Rostock, Germany; 3 Institute of Cell Biology and Biophysics, Leibniz University Hannover, 30419 Hannover, Germany; annegret.becker@gmx.de (A.B.); ngezahayo@cell.uni-hannover.de (A.N.); 4 Research Institute for Farm Animal Biology (FBN), Institute of Genome Biology, 18196 Dummerstorf, Germany; trakooljul@fbn-dummerstorf.de; 5 Division of Medicine, Medical Biology and Electron Microscopy Centre, University of Rostock, 18057 Rostock, Germany; marcus.frank@med.uni-rostock.de; 6 Department of Life, Light and Matter, University of Rostock, 18059 Rostock, Germany; 7 Comprehensive Cancer Center-Mecklenburg Vorpommern (CCC-MV), Campus Rostock, 18057 Rostock, Germany

**Keywords:** cell lines, prostate cancer, C-CPE, gold nanoparticle, GNOME-LP

## Abstract

Claudin (CLDN) proteins are commonly expressed in cancers and targeted in novel therapeutic approaches. The C-terminal of *Clostridium perfringens* enterotoxin (C-CPE) efficiently binds several claudins. In this study, recombinant C-CPE conjugated to gold nanoparticles (AuNPs) has been used for prostate adenocarcinoma (PAC) and transitional cell carcinoma (TCC) cell killing in vitro using gold-nanoparticle-mediated laser perforation (GNOME-LP). A PAC and TCC cell lines, as well as red fluorescence variants, allowing deep tissue imaging, were used. CLDN-3, -4, and -7 expression was confirmed by qPCR and immunofluorescences. The binding of C-CPE-AuNPs complexes on the cell surface was examined by scanning electron microscopy (SEM). Further, transcriptome analysis was carried out to evaluate the effect of C-CPE binder on the biological response of treated cells. Directed C-CPE-AuNP binding verified the capability to target CLDN receptors. Transcriptome analysis showed that C-CPE binding may activate immune and inflammatory responses but does not directly affect cell survival. Cancer cells ablation was demonstrated using a combination of GNOME-LP and C-CPE-AuNPs treatment reducing tumor cell viability to less than 10% depending on cell line. The fluorescent cell lines and the verified proof of concept in vitro provide the basis for perspective xenograft studies in an animal model.

## 1. Background

Prostate cancer is the second most frequently diagnosed cancer and the fifth leading cause of cancer-related death among men in 2020 [1,2]. The disease is at present incurable once it has metastasized, as metastases are highly resistant to current conventional therapies. Aside from humans, dogs are known to naturally develop prostate cancer [3]. In both species, adenocarcinomas of the prostate represent a locally invasive disease [4].

Histopathologically, prostate cancer can be classified into prostate adenocarcinoma (PAC) or transitional cell carcinoma (TCC) taking place in the bladder neck, urethra, or periurethral duct [2,5,6,7,8]. A great similarity is noted in the microscopic features and treatment response between invasive TCC in dogs and humans [9,10]. Furthermore, canine PAC showed similarity in histopathology, biological behavior, and treatment response to human metastatic castration-resistant prostate cancer (CRPC) [11,12,13,14]. Therefore, canine PAC and TCC are considered a reliable model for the testing of novel therapies for CRPC and bladder cancer in humans [7,11]. Consequently, data obtained in dogs may also lead to progress in human tumor research.

Since therapeutic options for CRPC and canine prostate cancer are limited to cytotoxic chemotherapy with unsatisfactory results, the development of novel treatments addressing specific molecular targets is required for both dogs and humans. In this context, different components of the cellular tight junctions have been moved in the spotlight [15,16].

Among promising tight-junction molecular targets for cancer diagnosis and therapy, claudins (CLDNs) are proteins abnormally regulated in different human and animal neoplasms affecting the mammary gland, prostate, pancreas, and colon [17,18,19,20,21,22,23]. The CLDN family consists of more than 20 proteins essential for tight-junction formation in epithelial and endothelial cells. Additionally, CLDNs are important regulators of paracellular transport and maintenance of cell polarity [17,23,24,25,26].

The second extracellular loop of CLDN-3, -4, and -7 acts as a receptor for the *Clostridium perfringens* enterotoxin (CPE) [27,28,29,30]. The C-terminal domain of CPE (C-CPE) by itself retains its high-affinity binding to CLDN but overcomes the toxic drawback of full-length CPE, which limited its use to local therapy. Considering C-CPE’s ability to modulate the tight-junction, and thus the barrier function of epithelium and endothelium in a noncytotoxic way, C-CPE has emerged as a promising therapeutic agent [31,32,33].

In recent years, laser therapy using gold nanoparticles (AuNPs) has rapidly evolved as a noninvasive thermotherapy for cancer as it enables hyperthermia of tumor tissues [34,35,36,37,38]. During laser treatment, AuNPs are delivered into tumors and are irradiated with laser light. AuNPs absorb light energy, causing electron excitation and subsequent nonradiative relaxation. The absorbed light is converted into heat, which irreversibly develops cell membrane disruption or protein denaturation of the surrounding tumor cells [35,39]. It is noteworthy that the laser does not need to be focused on the nanoparticles

Although AuNPs can passively accumulate in cancer cells [40], they have a nonspecific connection with cell membranes [39,41]. Due to the AuNPs accumulation in normal cells, an undesirable damage is associated with nontargeted AuNPs [42]. A previous study of our group confirmed that the C-CPE bound to cell lines expressing CLDN-3, -4, and -7 but was not able to target cells without CLDN-3, -4, and -7 expression. Furthermore, we demonstrated that C-CPE-AuNPs can be used to specifically and efficiently ablate different human cell lines expressing CLDN-3, -4, and -7 by gold-nanoparticle-mediated laser perforation (GNOME-LP) technique [43,44]. However, the used cell lines do not allow one to realize experimental in vivo experiments going for deep tissue imaging. In order to address this, stably transfected cell lines expressing red fluorescent proteins were established. As the genomic insertion of these marker proteins can affect the cellular response, the current study investigated claudin targeting in a prostate cancer in vitro model expressing red fluorescent marker proteins.

This study aimed to evaluate the elimination of stably transfected canine PAC and TCC tumor cell lines using C-CPE-AuNPs complex and GNOME-LP, and characterize, if the red fluorescent cell lines emission interferes with conventional laser ablation and optimized the therefore required parameters in vitro. Further, to investigate if the C-CPE binding affects the cell viability, a transcriptome analysis of the cells after treatment with C-CPE was performed.

## 2. Results

### 2.1. CLDN Gene Expression in Transfected Cell Lines

**Figure 1:**
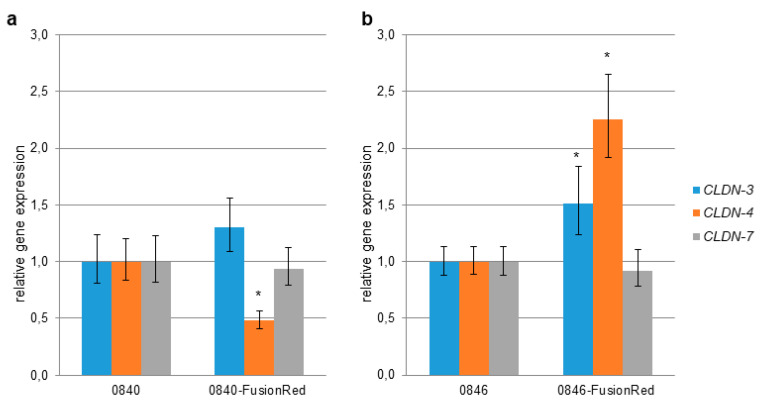
*CLDN-3*, *-4*, and *-7* gene expression in transfected tumor cell lines. Gene expression was measured via quantitative real-time RT-PCR, and results were normalized to the expression of *GAPDH* and *ACTB*. (**a**) *CLDN* gene expression in 0840-Fusionred in comparison to native 0840. (**b**) *CLDN* gene expression in 0846-Fusionred in comparison to native 0846. Error bars represent the mean ± standard deviation (SD). * *p* < 0.05 indicates statistically significant differential expression of *CLDN* compared to nontransfected cell lines.

Gene expression level of *CLDN-3*, *-4*, and *-7* in transfected cell lines were examined by quantitative real-time RT-PCR and compared to the native cell lines. The level of *CLDN-4* expression in 0840-FusionRed was significantly lower in comparison to the reference cell line (Figure 1). In contrast, the levels of *CLDN-3* and *-4* in 0846-FusionRed were higher than those of the reference cell line.

### 2.2. CLDN Protein Immunofluorescence

**Figure 2:**
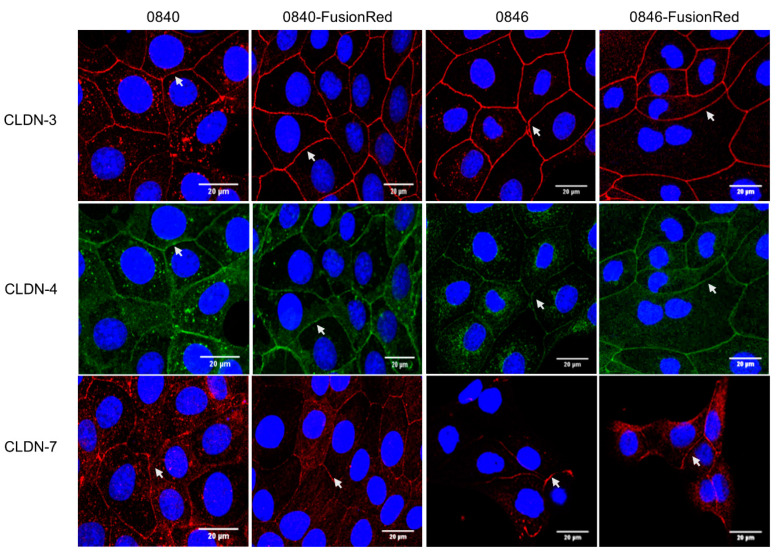
Expression of CLDNs protein in native and transfected tumor cell lines. Cells were subjected to immunostaining with corresponding antibodies using fluorescein-conjugated secondary antibodies, showing red signals for CLDN-3 and -7 and green signals for CLDN-4. DAPI was used for blue nuclei staining. Images were observed under confocal microscopy. Arrows indicate CLDN localization on cell–cell contact.

The presence of CLDN-3, -4, and -7 proteins in native and transfected cell lines were subsequently examined by immunostaining. In the native 0840 cell line, CLDN-3, -4, and -7 proteins showed a strong signal and were localized at the cell membranes and in the cytoplasm (Figure 2). For the 0840-FusionRed cell line, CLDN-3, -4, and -7 proteins were found at the cell membrane; CLDN-7 was weakly expressed (Figure 2).

Expression of CLDN-3 and -7 proteins in the native 0846 cell line was localized at the cell membranes, whereas CLDN-4 was punctually localized in the cytoplasm and at the cell membrane. In the generated 0846-FusionRed cell line, CLDN-3, -4, and -7 proteins were strongly distributed along the cell membranes (Figure 2).

### 2.3. Binding of C-CPE to Cell Lines

**Figure 3:**
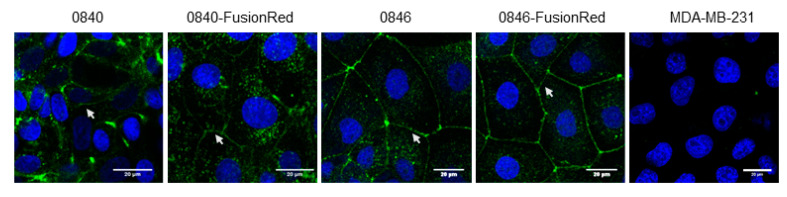
Binding of C-CPE to CLDN. Specific binding of C-CPE-Strep-Tactin Chromeo 488 complex on CLDN expressing cells at cell–cell contact (green); note that C-CPE did not bind onto cells such as MDA-MB-231 that do not express CLDNs. Images were observed under confocal microscopy. Arrows indicate C-CPE binding on cell–cell contact.

C-CPE’s capability to target CLDN was determined through the visualization of C-CPE-CLDN binding. The C-CPE-Strep-Tactin Chromeo 488 complex was detected along cell membranes of native and transfected cell lines at cell–cell junction between adjacent cells (Figure 3).

### 2.4. Electron Microscopy

**Figure 4:**
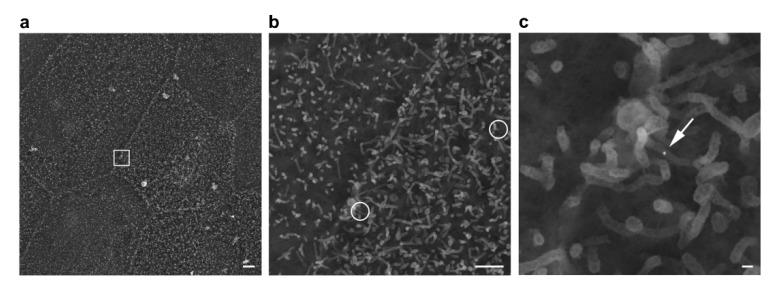
Scanning electron microscopy of C-CPE-AuNPs on a 0846-FusionRed cell. (**a**) 2000× overview of the cell culture and stepwise higher magnification of a cell–cell border area presented in (**b**) 10,000× with AuNPs encircled and (**c**) 40,000× detailing a 25 nm AuNP (arrow) on surface microvilli. The white box outlined in (**a**) indicates the area magnified in (**c**). Scale bars are 2 µm, 1 µm, and 100 nm, respectively.

To investigate the binding of AuNPs and C-CPE-AuNPs complexes on the cell surface, 0846 and transfected 0846-FusionRed cells were examined by SEM. AuNPs, appearing as white bright spheres in high-resolution SEM analysis, were detected, e.g., on microvilli extending from the cell surfaces of 0846 and 0846-FusionRed cells (Figure 4). Uncoupled AuNPs showed a broad distribution at the cell surface, while C-CPE AuNPs were found primarily located in close distance to cell–cell borders. Whereas the presence of AuNPs and C-CPE-AuNPs on microvilli may indicate nonspecific surface binding, the internalization of AuNPs was clearly visible by SEM in some areas (see Supplementary File S1).

### 2.5. Comparative Analysis of Differentially Expressed Genes (DEGs) between C-CPE-Treated and Nontreated Cell Lines

Differential expression analysis revealed several genes that were differentially regulated in C-CPE-treated cells when compared with nontreated cells with a false discovery rate (FDR) < 0.05. The comparison analysis of 0840-C-CPE vs. 0840 showed only 11 DEGs. Contrarily, 0840-FusionRed-C-CPE and 0840-FusionRed revealed 1070 DEGs.

**Figure 5:**
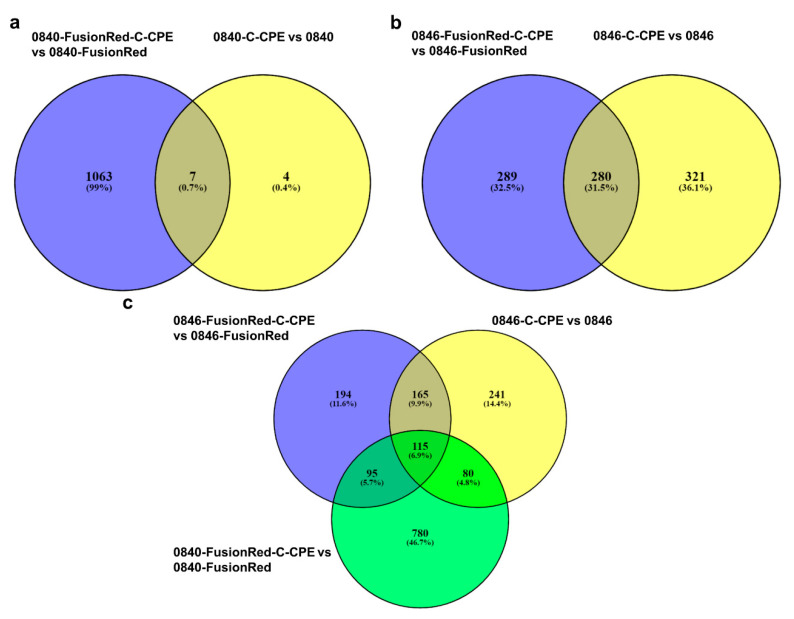
Venn diagram of dysregulated gene in C-CPE-treated cell lines in comparison to nontreated cells. (**a**) Seven DEGs shared between 0840-C-CPE vs. 0840 and 0840-FusionRed-C-CPE vs. 0840-FusionRed. (**b**) 280 DEGs shared between 0846-C-CPE vs. 0846 and 0846-FusionRed-C-CPE vs. 0846-FusionRed. (**c**) Overlapping DEGs between 0846-C-CPE vs. 0846, 0846-FusionRed-C-CPE vs. 0846-FusionRed, and 0840-FusionRed-C-CPE vs. 0840-FusionRed cells.

A total of seven DEGs were common among 0840-C-CPE vs. 0840 and 0840-FusionRed-C-CPE vs. 0840-FusionRed (Figure 5a).

Further, the comparison analysis of 0846-C-CPE to 0846 demonstrated 601 DEGs. In total, 569 genes were extracted from the comparison of 0846-FusionRed-C-CPE to 0846-FusionRed. Additionally, the comparison between 0846-C-CPE vs. 0846 and 0846-FusionRed-C-CPE vs. 0846-FusionRed revealed 280 overlapping genes (Figure 5b). Only one DEG was shared among all treated cell lines, whereas a total of 115 DEGs were shared among 0846-C-CPE vs. 0846, 0846-FusionRed-C-CPE vs. 0846-FusionRed, and 0840-FusionRed-C-CPE vs. 0840-FusionRed cells (Figure 5c).

### 2.6. Functional and Pathway Enrichment Analysis of DEGs of C-CPE-Treated Cell Lines

In order to evaluate the effect of C-CPE on the gene expression independent from cell lines, GO and KEGG pathways analyses were carried out for overlapping DEGs between 0846-C-CPE vs. 0846, 0846-FusionRed-C-CPE vs. 0846-FusionRed, and 0840-FusionRed-C-CPE vs. 0840-FusionRed cells.

The analyses could not be performed for 0840-C-CPE vs. 0840 as only 11 DEGs were detected.

GO enrichment analysis revealed that the overlapping DEGs are involved in a number of biological processes (BP) including inflammatory response, immune response, and cellular response to interleukin-1. In terms of cellular components (CC), DEGs were mostly enriched in extracellular space, integral component of plasma membrane, and external side of plasma membrane. Molecular functions (MF) analysis indicated that the overlapping DEGs were only associated with chemokine activity (Table 1). Subsequent KEGG pathway analysis revealed that overlapping DEGs enriched in KEGG pathways are related to signaling molecules and interaction, signal transduction, infectious and immune disease, and cancer (Table 2).

### 2.7. Selective Cancer Cells Ablation Using GNOME-LP and C-CPE-AuNPs Complex

**Figure 6:**
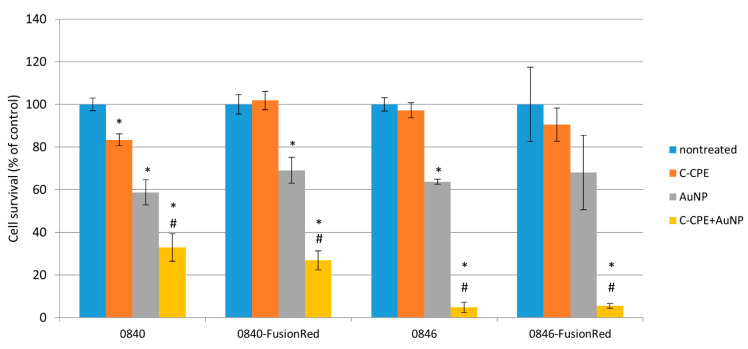
GNOME-LP-mediated tumor cell killing using C-CPE functionalized AuNPs. Increased cell killing efficiency of GNOME-LP in presence of C-CPE-AuNPs compared to GNOME-LP in combination with nonfunctionalized AuNPs. SYTOX green uptake was used as an indicator of cell death after GNOME-LP application. The graph represents the mean ± standard deviation (SD) of cell survival relative to untreated cells as a control reference. Significant differences to untreated controls were analyzed with Student’s test. *: *p* < 0.05, # Significant difference to cells treated with AuNPs only; *p* < 0.05.

Laser exposure of 0840 native and transfected CLDN expressing cells in combination with C-CPE functionalized AuNPs significantly reduced number of vital cells down to 32.73% and 26.86%, respectively, in comparison to untreated cells (Figure 6). GNOME-LP in combination with C-CPE functionalized AuNPs significantly decreased cell survival to 8.55% and 5.52% in native and transfected 0846 cell lines, respectively. In cells treated with C-CPE alone, GNOME-LP application did not significantly impair cell survival. The application of GNOME-LP in the presence of nonfunctionalized AuNPs reduced the number of 0840 cells to 41.56% and number of 0840-FusionRed cells to 30.91%. Similarly, laser exposure of 0846 in combination with nonfunctionalized AuNPs significantly decreased cells survival to 69.81%. Killing efficiency in the presence of functionalized AuNPs (C-CPE-AuNPs) was significantly higher in comparison to killing with nonfunctionalized AuNPs. Cancer cell killing after GNOME-LP treatment was quantified by Hoechst and SYTOX staining where SYTOX green uptake was used as an indicator of cell death.

## 3. Discussion

In vivo models are the key to understanding the pathogenesis of cancer and the development of novel therapeutic approaches [45]. Although in vitro systems offer several possibilities for basic drug evaluation, they remain limited for the evaluation of complex interactions. Accordingly, xenograft models derived by cell line injections are of considerable value. However, these models—if not transfected with a special marker—hardly allow for the characterization of early implantations phases and early tumor development [46]. Herein, two canine cancer cell lines were used, 0840-FusionRed and 0846-FusionRed, both stably expressing red fluorescent protein allowing deep tissue imaging in perspective [47].

It is well documented that CPE receptors CLDN-3, -4, and/or -7 are abnormally regulated in many tumor types including prostate cancer [15,17,18,19,20,48], which also was confirmed for the used 0840 and 0846 cell lines [49]. In the present study, *CLDN-3, -4, and -7* expressions in generated fluorescent cell line 0840-FusionRed revealed no significant difference in comparison to native 0840 for *CLDN-3* and *-7*; however, *CLDN-4* was significantly decreased. An analysis of *CLDN-7* in 0846-FusionRed showed no difference in expression, whereas *CLDN-3* and *-4* were even higher expressed after transfection. Additional immunofluorescence staining confirmed strong expression of all three CLDN proteins in all used cell lines. Therefore, 0840-FusionRed appears as a sufficient model for further experiments despite significant decrease in *CLDN-4* mRNA level measured by qPCR. Interestingly, immunostaining revealed that CLDN-3, -4, and -7 in 0840 cells, as well as CLDN-4 in 0846, were punctually located in the cytoplasm. Such apparent miss-localizations were also described for the CLDN-4 protein in human prostate cancer-derived cell lines and may be related to the loss of cellular organization due to a defect in tight-junction formation or cell polarity—features common in tumor cells [50].

The binding of CPE to CLDN-3 and -4 can trigger cell death [51,52,53,54]. Therefore, it was proposed to use CPE for tumor therapy. However, studies in vivo revealed that the systematic administration of full-length CPE in mice was toxic and thus limited its use to local therapies [52]. Our previous published study demonstrated that the noncytotoxic C-terminal domain of CPE, which preserves CPE’s binding affinity to CLDN receptors, is capable of functionalizing AuNPs. The imaging of C-CPE binding to the canine tumor cell lines proved that the protein can specifically target CLDN-3, -4, and -7, demonstrating that the functionalization did not alter the binding capacity to CLDN [43].

To confirm the specific binding of the functionalized AuNPs, scanning electron microscopy was performed in the present study. These images indicated that the C-CPE conjugated AuNPs retain the affinity to its receptors (CLDN-3, -4, and -7) on 0846 and 0846-FusionRed cell lines. The GO term and KEGG pathways analyses of DEGs demonstrated significant differences between C-CPE-treated and nontreated cell lines. These changes were mostly related to the cell surface/membrane as expected. C-CPE binding can disrupt the tight-junctional barrier but does not have a cytotoxic effect [29]. Furthermore, transcriptome analysis revealed that the C-CPE binding to the cell lines enhances immune responses. However, the Go term and a KEEG pathways analysis revealed no induction of apoptosis or necrosis in which C-CPE binding itself was detectable.

The GNOME-LP technology has been used for the cellular introduction of dyes as well as siRNA into different cell types via transient cell permeabilization [55,56,57,58]. The present report shows that C-CPE coupled to Strep-Tactin conjugated AuNPs in combination with GNOME-LP technique can be used for specific targeting of CLDNs expressing tumor cell lines.

A previous study of our group showed that the energy power of the applied laser at 60 mJ/cm^3^ and a scanning speed of 0.5 cm/s in combination with C-CPE-AuNPs reduced cell survival to less than 30% of claudin expressing cell lines [43]. In a first experiment, GNOME-LP with the same settings accordingly reduced cell survival to about 30% in native 0846 cells but showed no effect on the transfected fluorescence cells (see Supplementary File S2). At 532 nm (laser wavelength), the red fluorescent dye FusionRed has approximately 50% absorption (50% of dye molecules absorb light at 532 nm). Therefore, depending on dye concentration in the cells, a significant amount of laser light might be absorbed, thus reducing the overall effect on AuNPs. Therefore, GNOME-LP was applied at the maximal laser fluence (72 mJ/cm^3^) on native and fluorescent cell lines. Using the new setting, GNOME-LP in combination with C-CPE functionalized AuNPs reduced cell survival to down to 30% in 0840 and less than 10% in 0846 (native and fluorescent) cells.

The significant killing of 0840 (native and transfected) and native 0840 cells treated with nonfunctionalized AuNPs may be related to endocytosis activity, allowing them to internalize the AuNPs. This interpretation is supported by SEM analysis showing the presence of many uncoupled AuNPs that are bound nonspecifically on the cell surface microvilli even after three hours of incubation whereas fewer C-CPE functionalized AuNPs are present on the cell surface, mostly restricted along cell–cell borders. This suggests that C-CPE-AuNPs efficiently bind to their protein targets and are rapidly internalized into the cells as it can be traced through SEM (see Supplementary File S1). However, the results show that the functionalization of AuNPs with C-CPE increases the ablation efficiency of CLDN expressing tumor cell lines in comparison to cells treated only with AuNPs.

The results of this study confirm for the first time that the therapy concept of C-CPE functionalized AuNPs can be used efficiently against PAC and TCC cell lines. By using GNOME-LP system and C-CPE functionalized AuNPs, an irreversible laser ablation of prostate tumor cells was achieved in vitro. Cells, which were irradiated with maximal laser power without C-CPE-AuNPs, maintained viability. Likewise, cells incubated with C-CPE and irradiated with the maximal laser fluence maintained viability as well. A combination of laser treatment and C-CPE-AuNPs, however, reduced tumor cell viability down to less than 10% in 0846.

To further extend the presented in vitro findings, in vivo studies need to be carried out as the next step. The same cell lines used for the in vitro findings could be detectable in vivo through deep tissue imaging, thereby enabling one to observe tumor growth and subsequently possible tumor ablation through C-CPE treatment in a living animal. In vivo studies could allow the characterization if the C-CPE complex is able to diffuse through the extracellular matrix and bind to tumor tissues. If successful, a combination between GNOME-LP and functionalized AuNPs may establish a treatment option for canine PAC and TCC cancer.

## 4. Materials and Methods

### 4.1. Cell Lines and Culture

Canine tumor cell lines TihoDTCC0840 (0840) and TihoDProAdCarc0846 (0846) were previously derived by our group from canine prostate carcinomas [59,60]. Both cell lines have been demonstrated to express CLDN-3, -4, and -7 [49].

Transfected cell lines 0840-FusionRed and 0846-FusionRed were generated and characterized by our group [47]. Cell line 0840 was derived from a transitional cell carcinoma (TCC), whereas 0846 was derived from prostate adenocarcinoma (PAC) tissue.

The cells were cultivated separately in 25 cm^2^ cell culture flasks in medium 199 (Gibco by Life Technologies, Darmstadt, Germany), supplemented with 10% fetal calf serum (FCS Superior, Biochrom GmbH, Berlin, Germany) and 2% penicillin/streptomycin (Biochrom GmbH, Berlin, Germany), and incubated in a humidified incubator maintained at 37 °C with 5% CO_2_. Cultivation medium was replaced twice per week.

### 4.2. RNA Isolation and cDNA Synthesis

Total RNA was isolated from transfected and native prostate tumor cell lines using the RNeasy^®^Mini Kit RNA Purification (Qiagen, Hilden, Germany) according to the manufacturer’s instruction. RNA isolation was performed three times per cell line. DNase digestion was carried out with RNase-Free DNase Set (Qiagen) to avoid genomic DNA contamination. Subsequently, cDNA synthesis was performed using Transcriptor First Strand cDNA Synthesis Kit (Roche, Mannheim, Germany), 1000 ng of total RNA, and anchored-oligo (dt)_18_ primer according to the manufacturer’s instructions.

### 4.3. Quantitative Real-Time RT-PCR

To verify expression of *CLDN* genes after transfection process, transfected and native prostate tumor cell lines were comparatively analyzed by quantitative PCR. Primer pairs for *CLDN-3, CLDN-4,* and *CLDN-7* were designed according to the mRNA sequences given by the National Center for Biotechnology Information (NCBI) (Table 3). Real-time PCR was performed using Fast SYBR™ Green Master Mix Kit (Life Technologies, Darmstadt, Germany) according to the manufacturer´s instructions. Quantitative PCR reactions were carried out in real-time PCR cycler peqSTAR 96q (PEQLAB Biotechnologies GmbH, Erlangen, Germany). The qPCR results were analyzed using the delta-delta CT (ΔΔCT) method relative to nontransfected cells. Mean values of three wells were used per measured gene. Normalization was performed against the two housekeeping genes, beta-actin (*ACTB*) and glyceraldehyde 3-phosphate dehydrogenase (*GAPDH*). The experiment was performed three times.

### 4.4. Immunofluorescence Assay

Immunofluorescence was performed for native and transfected cell lines to further confirm CLDN expression. The cells were cultivated on rat collagen type I (Trevigen, Gaithersburg, MD, USA) coated glass coverslips. Thereafter, cells were washed with PBS, fixed wit (1:1) Acetone/Methanol for 5 min at −20 °C and blocked for 30 min with 1% BSA (bovine serum albumin, Sigma-Aldrich, Taufkirchen, Germany) in PBS at 37 °C. CLDNs were stained with primary antibodies (Table 4) diluted in PBS containing 1% BSA, overnight at 4 °C. Cells were washed with PBS. iFlour™ 488 antimouse (AAT Bioquest, Sunnyvale, CA, USA) and iFlour™ 555 antirabbit (AAT Bioquest) were diluted 1:500 in PBS containing 1% BSA and added to the respective cells as secondary antibodies for 1 h at 37 °C. For nuclei staining, DAPI (2 µM) (Sigma-Aldrich) was used. Cells were stored in PBS at 4 °C for further analysis. As a control for unspecific binding sites, cells were also incubated with only the secondary antibodies. Fluorescent images of cells were taken with a Nikon Eclipse TE2000-E confocal laser scanning microscope (400 nm for DAPI, 555 nm for CLDN-3 and -7 proteins, and 488 nm for CLDN-4), with a 60× water immersion objective and software EZ-C1 3.80 (Nikon, Düsseldorf, Germany).

### 4.5. Visualization of C-CPE-CLDN Binding

The *C. perfringens* enterotoxin C-terminal fragment (C-CPE) with an N-terminal Strep-tag II was prepared as described previously [43]. MDA-MB-231 cell line was used as negative control since it does not express CLDN-3, -4, and -7.

For C-CPE-CLDN binding visualization, the C-CPE was conjugated to green-fluorescent Strep-Tactin^®^ Chromeo 488 dye (IBA, Goettingen, Germany). The complex was freshly generated before usage, by mixing 2.5 μL Strep-Tactin^®^ Chromeo 488 (0.5 mg/mL) as recommended by the manufacturer with C-CPE dissolved in elution buffer. The mix was incubated overnight at 4 °C to allow binding of C-CPE with Strep-Tactin^®^ Chromeo 488. To reach a final concentration of 20 μg/mL C-CPE, the mixture was diluted with 250 µL culture medium. Cells were cultured in a monolayer and stained for 3 h at 37 °C with 20 µg/mL C-CPE-Chromeo 488 complex. For nuclei staining, 1 µM Hoechst 33258 (Sigma-Aldrich) was used. Thereafter, cells were fixed with 4% formaldehyde for 10 min at room temperature and stored in PBS at 4 °C. The cells were imaged with a Nikon Eclipse TE2000-E confocal laser scanning microscope (346 nm for Hoechst 33258 and 488 nm for Chromeo 488) with a 60× water immersion objective and software EZ-C1 3.80 (Nikon).

### 4.6. Electron Microscopy

To examine the binding of AuNPs and C-CPE-AuNPs on cell surfaces, the 0846 and transfected 0846-FusionRed cell lines were analyzed with SEM. Confluent cells were treated with AuNPs and C-CPE-AuNPs for 3 h in a cell culture incubator to allow complex adhesion to the cells. The cells were exposed to a pulsed laser with 60 mJ/cm² at a scanning speed of 0.5 cm/s. Subsequently, the cells were fixed with 4% formaldehyde, washed with PBS, and stored for further processing. For SEM preparation, the coverslips were dehydrated with a graded series of ethanol completed with an acetone step prior to critical point drying with CO_2_ as an intermedium (Emitech K850 critical point dryer, Emitech/Quorum Technologies Ltd., Laughton, UK). The coverslips were flat-mounted on SEM-stubs with adhesive carbon tape (Plano, Wetzlar, Germany) and coated with a carbon layer (Leica SCD500, Leica Microsystems, Wetzlar Germany). Specimens were analyzed in a field-emission SEM (Zeiss Merlin VP compact, Carl Zeiss Microscopy, Oberkochen, Germany) equipped with HE-SE and in-lens-Duo detectors operated at 5 kV and images with a size of 1024 × 768 pixels were recorded at different steps of magnification.

### 4.7. Treatment with C-CPE for Sequencing

C-CPE was prepared as described previously [43]. Native and transfected cell lines were seeded in triplicate with a density of 5 × 10^5^ cells in 6-well plates 48 h to reach monolayer. C-CPE with a concentration of 20 µg/mL was added, and the cells incubated for 3 h to allow binding of C-CPE to CLDNs. In the next step, culture medium was removed, and cells were washed with 5 mL phosphate buffered saline (PBS). TrypLE^TM^ Express (Gibco by Life technologies^TM^, Darmstadt, Germany) was used for detaching cells, centrifugation at 1000 rpm for 10 min followed for pelleting. Pellets were stored at −80 °C and followed by RNA-isolation for transcriptome analysis.

### 4.8. RNA Isolation and Library Generation

Total RNA was isolated from transfected and native prostate tumor cell lines with C-CPE treatment and without C-CPE treatment using the RNeasy^®^Mini Kit RNA Purification (Qiagen, Hilden, Germany) according to the manufacturer´s instruction. RNA isolation was performed three times per cell line. On-column DNase digestion was carried out with RNase-Free DNase Set (Qiagen Hilden, Germany) to avoid genomic DNA contamination.

The RNA quality was assessed using an Agilent RNA 6000 Nano kit and 2100 Bioanalyzer (Agilent). Samples with RNA integrity number (RIN) > 8 were used for the DNA library preparation using a TruSeq Stranded mRNA Sample Preparation kit according to the manufacture’s protocol (Illumina). In brief, 1 µg of total RNA was used as input for an mRNA enrichment using poly-T oligo coated magnetic beads and chemically fragmented under elevated temperature. The fragmented RNA was then reverse-transcribed into the first- and second-strand cDNA using random hexamers and Superscript II reverse transcriptase. Double-stranded cDNA fragments were ligated with TruSeq RNA adapters with a unique DNA sequencing index and PCR-amplified. The DNA libraries were quality-controlled using an Agilent Technologies 2100 Bioanalyzer and Agilent DNA-1000 Chip kit.

### 4.9. RNA Sequencing

cDNA library concentration was quantified using a Qubit dsDNA HS Assay kit (Life technologies, Darmstadt, Germany) and normalized to 2 nM prior to multiplexing. The DNA libraries were sequenced at a final concentration of 13 pM for 125 bp single-end reads using the high-output mode on a HiSeq2500 (Illumina) at the sequencing facility of Genome Biology Institute, Leibniz Institute for Farm Animal Biology (FBN), Dummerstorf, Germany.

### 4.10. Data Processing and DEGs Analysis

The raw fastq reads were quality-checked using FastQC (version 0.11.5) (http://www.bioinformatics.babraham.ac.uk/projects/fastqc/, accessed on 1 May 2021) and preprocessed by filtering out low-quality reads with a mean Q-score < 20 and trimming adapter-like sequences using TrimGalore version 0.6.5. High-quality reads were aligned to the reference genome CanFam2 (Ensembl release 100) using Hisat2 version 2.2 [61,62]. Uniquely mapped reads to each gene were extracted from the HISAT2 mapping results using HTSeq version 0.8.0 [63]. The resulting RNA-seq gene count data were further analyzed for DEGs using edgeR package.

GO and KEGG analyses were applied for the functional annotation and pathway analysis using the Database for Annotation Visualization and Integrated Discovery (DAVID; https://david.ncifcrf.gov/, accessed on 1 May 2021). A list including all sequenced genes was used as background.

### 4.11. Tumor Cells Ablation by GNOME-LP and C-CPE-AuNPs Complex Interaction

For tumor cell killing using a laser beam, confluent cells in 96-wells were treated with the C-CPE-AuNPs complex for 3 h in a cell culture incubator to allow adhesion of the complex onto the cells. The C-CPE-AuNPs complex was generated as previously described [46,47]. Shortly, the Strep-tagged C-CPE in elution buffer and Strep-Tactin^®^ conjugated AuNPs (diameter 25 nm) (Aurion, Wageningen, Netherlands) were mixed and incubated overnight at 4 °C. The concentration was adjusted to 20 μg/mL C-CPE and 2.5 × 10^10^ AuNPs/mL with cell culture medium.

In addition to nontreated cells, cells incubated with either nonfunctionalized AuNPs or C-CPE alone were used as controls. Cells were exposed to a pulsed laser with 72 mJ/cm² at a scanning speed of 0.5 cm/s. Laser-treated cells were incubated for 30 min with 1 µM Hoechst 33258 (Sigma-Aldrich) and 10 min with SYTOX green (1:500, PromoCell GmbH, Heidelberg, Germany) in a cell incubator. Under the Ti-E inverted fluorescence microscope (Nikon, Duesseldorf, Germany), images were taken with 4x objective and Nikon Software Nis-Elements 4.4 (346 nm for Hoechst 33258 and 488 nm for SYTOX green). Vital cells were indicated by Hoechst uptake, whereas dead cells were referred by SYTOX and Hoechst uptake. Mean values of three wells were used per experiment. The experiment was performed three times. For cell survival quantification, Hoechst and SYTOX stained cells were counted with the image processing software ImageJ/Fiji V2.0.0 (Dresden, Germany).

### 4.12. Statistical Analysis

The results are given as the mean of at least three independent experiments for quantitative real-time RT-PCR and cell killing using GNOME-LP. Statistical analysis was performed using SAS software 7.1 (SAS Institute Inc., Cary, NC, USA). Significant differences in gene expression of CLDN-3, -4, and -7 were calculated using Student’s two-sided *t*-test. Statistical analysis of cell killing using GNOME-LP was performed using Dunnett´s multiple comparison test and Student´s two-sided *t*-test. Differences were considered statistically significant for *p* < 0.05.

## 5. Conclusions

In summary, the fluorescent cell lines and the verified proof of concept in vitro provide the basis for perspective xenograft in vivo studies.

Since dogs represent a model for prostate cancer, the development of therapeutic strategies provides an important contribution to translational research directed to treat humans, thus providing a benefit for both species.

## Figures and Tables

**Table 1:** GO analysis of C-CPE depending DEGs. Gray font represents a subclass relationship.

ID	Term	Count	FDR
Biological process			
GO:0006954	inflammatory response	11	6.43 × 10^−7^
GO:0071347	cellular response to interleukin-1	7	1.70 × 10^−5^
GO:0071356	cellular response to tumor necrosis factor	6	1.70 × 10^−3^
GO:0070098	chemokin × 10−mediated signaling pathway	4	1.09 × 10^−2^
GO:0071222	cellular response to lipopolysaccharide	8	2.29 × 10^−5^
GO:0006955	immune response	11	6.43 × 10^−7^
GO:0045766	positive regulation of angiogenesis	6	1.02 × 10^−2^
GO:0043491	positive regulation of protein kinase B signaling	5	4.14 × 10^−2^
Cellular components			
GO:0005615	extracellular space	17	1.06 × 10^−5^
GO:0009897	external side of plasma membrane	6	2.50 × 10^−2^
GO:0009986	cell surface	8	3.59 × 10^−2^
GO:0005887	integral component of plasma membrane	10	3.59 × 10^−2^
Molecular functions			
GO:0008009	chemokine activity	4	1.98 × 10^−2^

**Table 2:** KEGG pathway of C-CPE depending DEGs.

Class	ID	Term	Count	FDR
Signaling molecules and interaction	cfa04060	Cytokin × 10−cytokine receptor interaction	11	8.4 × 10^−7^
Signal transduction	cfa04668	TNF signaling pathway	11	8.4 × 10^−7^
	cfa04064	NF-kappa B signaling pathway	10	8.4 × 10^−7^
	cfa04010	MAPK signaling pathway	9	8.5 × 10^−3^
Infectious disease	cfa05132	Salmonella infection	5	3.2 × 10^−2^
	cfa05140	Leishmaniasis	5	1.3 × 10^−2^
	cfa05166	HTLV-I infection	9	1.2 × 10^−2^
	cfa05142	Chagas disease	7	2.6 × 10^−3^
	cfa05133	Pertussis	6	2.6 × 10^−3^
Immune disease	cfa05323	Rheumatoid arthritis	9	1.6 × 10^−6^
	cfa05321	Inflammatory bowel disease (IBD)	4	3.6 × 10^−2^
Development and regeneration	cfa04380	Osteoclast differentiation	8	1.1 × 10^−3^
immune system	cfa04620	Toll-like receptor signaling pathway	6	8.5 × 10^−3^
	cfa04621	NOD-like receptor signaling pathway	5	8.5 × 10^−3^
cancer	cfa05200	Pathways in cancer	10	2.7 × 10^−2^

**Table 3:** Primer sequences used for real-time PCR.

Target Gene	Forward Primer Sequence	Reverse Primer Sequence	Accession Number
*CLDN-3*	5′ gcccaccaagatcgtctact 3′	5′ gtctggagtgggttggtctc 3′	NM_001003088.1
*CLDN-4*	5′ gcctcacttacccacctgac 3′	5′ accagtttgtggcaccttca 3′	XM_005620962.3
*CLDN-7*	5′ cacgatgggcatgaagtgta 3′	5′ taccaaggcagcaagacctc 3′	XM_546584.5
*ACTB*	5′ tcgctgacaggatgcagaag 3′	5′ gtggacagtgaggccaggat 3′	NM_001195845.2
*GAPDH*	5′ cagtatgattctacccacggcaa 3′	5′ cctggaagatggagatggactt 3′	NM_001003142.2

**Table 4:** Primary antibodies used for immunofluorescence assays.

Protein	Antibody	Concentration
CLDN-3	Rabbit antimouse CLDN-3 34-1700 (Thermo Fischer Scientific, Waltham, MA, USA)	3 µg/mL
CLDN-4	Mouse antihuman CLDN-4 34-1700 (Thermo Fischer Scientific)	3 µg/mL
CLDN-7	Rabbit antihuman CLDN-7 32-9400 (Thermo Fischer Scientific)	2 µg/mL

## Supplementary Materials

The supplementary material is available online at https://www.mdpi.com/article/10.3390/ijms222212289/s1.

## Abbreviations

CLDN: Claudin; C-CPE: C-terminus of Clostridium perfringens enterotoxin; AuNPs: Gold nanoparticles; PAC: Prostate adenocarcinoma; TCC: Transitional cell carcinoma; GNOME-LP: Gold-nanoparticle-mediated laser perforation; SEM: Scanning electron microscopy; RFP: Red fluorescent protein; G418: Geneticin.
