# Supplementary figures and images for: Ablation of Red Stable Transfected Claudin Expressing Canine Prostate Adenocarcinoma and Transitional Cell Carcinoma Cell Lines by C-CPE Gold-Nanoparticle-Mediated Laser Intervention
Source: Int J Mol Sci. 2021 Nov 13;22(22):12289. doi: 10.3390/ijms222212289 (PMC8618062; doi:10.3390/ijms222212289)

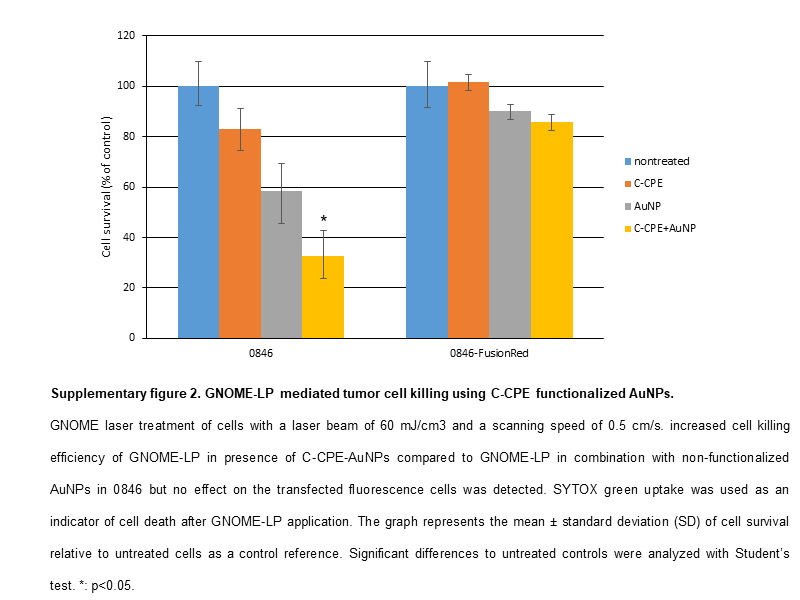

Supplement: Supplementary file 1 [file ijms-22-12289-s001.zip › Figure S2.tif]

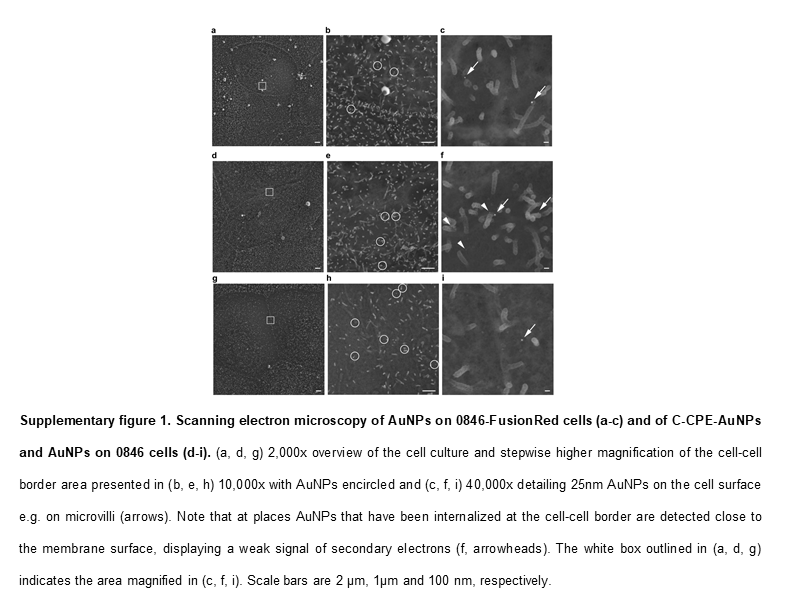

Supplement: Supplementary file 1 [file ijms-22-12289-s001.zip › Figure. S1.tif]
